# Salvage hemostasis with endoscopic suturing for refractory transverse colonic ulcer bleeding

**DOI:** 10.1055/a-2695-4580

**Published:** 2025-09-18

**Authors:** Tung-Lung Wu, Pei-Yuan Su, Yang-Yuan Chen, Hsu-Heng Yen

**Affiliations:** 1 Division of Gastroenterology, Changhua Christian Hospital, Changhua, Taiwan; 2 Department of Post-Baccalaureate Medicine, College of Medicine, National Chung Hsing University, Taichung, Taiwan; 3 Artificial Intelligence Development Center, Changhua Christian Hospital, Changhua, Taiwan

Gastrointestinal bleeding is a common medical emergency, with most cases effectively managed using standard endoscopic therapies such as epinephrine injection, mechanical clipping, and thermal coagulation. However, in cases where bleeding recurs despite endoscopic treatment, escalation to rescue strategies such as angiographic embolization or surgery becomes necessary. Endoscopic suturing has recently emerged as a salvage hemostatic technique before surgery 1 2 , complementing its established roles in endoscopic gastroplasty 3 and wound closure 4 . We report a case of recurrent colonic ulcer bleeding in the transverse colon that was successfully treated using an endoscopic suturing system after the failure of conventional endoscopic therapies.

A 62-year-old woman with acute myeloid leukemia presented with spontaneous, recurrent bleeding from an ulcer in the proximal transverse colon. Over 2 weeks, seven endoscopic hemostasis attempts – epinephrine injection, clipping, and thermal coagulation – failed to achieve durable control ( Fig. 1 ). Consequently, the OverStitch NXT system (Boston Scientific, Marlborough, Massachusetts, USA) was employed as salvage therapy following shared decision making with the patient as a final endoscopic option prior to surgery. The OverStitch device was mounted onto an Olympus GIF-H290 endoscope (Olympus, Tokyo, Japan), which was carefully advanced to the ulcer. A stitch was placed through healthy mucosa at the ulcer margin, and a continuous running suture approximated the defect ( Video 1 ). A single cinch was then deployed to secure the suture. Endoscopy performed 3 days later confirmed complete edge approximation and hemostasis ( Fig. 2 ).

**Fig. 1:**
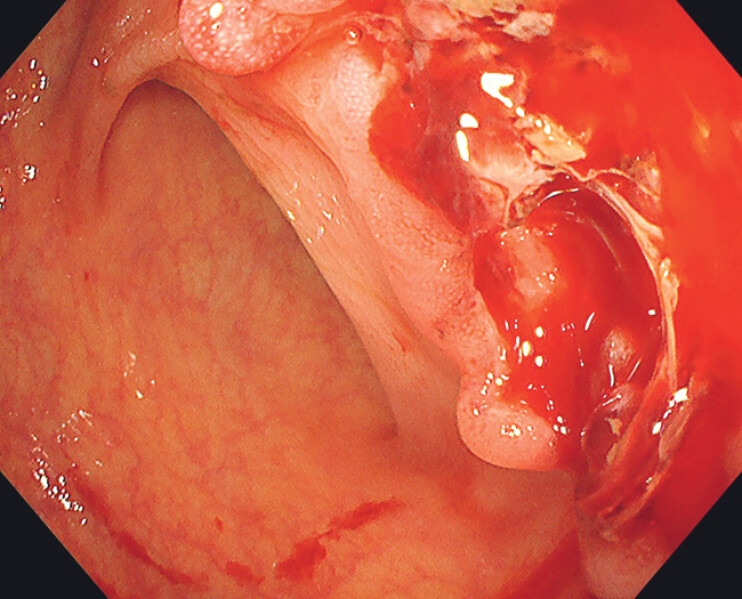
A bleeding ulcer in the transverse colon documented 1 day before suturing; over the preceding 2 weeks, seven attempts at endoscopic hemostasis had been performed.

Endoscopic suturing achieved definitive hemostasis in colonic ulcer bleeding refractory to conventional therapy, offering a potential salvage option for lower gastrointestinal hemorrhage before surgical intervention.Video 1

**Fig. 2:**
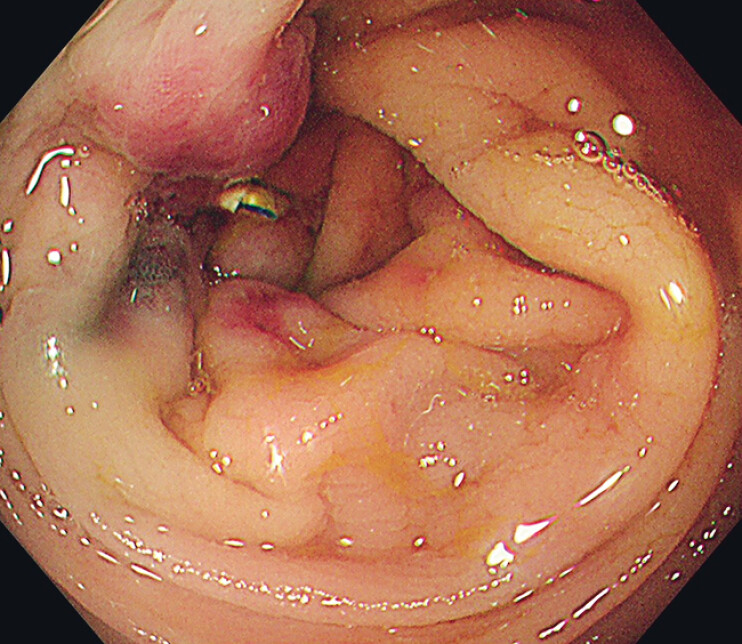
After endoscopic suturing of the ulcer, endoscopy on Day 3 confirmed complete edge approximation and hemostasis.

At the 2-week follow-up, the patient had resumed normal oral intake and had normal bowel function without further bleeding.

This case highlights endoscopic suturing as a viable option for managing recurrent colonic ulcer bleeding when conventional endoscopic methods fail.

Endoscopy_UCTN_Code_TTT_1AQ_2AZ

## Notes

Endoscopy E-Videos https://eref.thieme.de/e-videos
*E-Videos* is an open access online section of the journal *Endoscopy* , reporting on interesting cases and new techniques in gastroenterological endoscopy. All papers include a high-quality video and are published with a Creative Commons CC-BY license. Endoscopy E-Videos qualify for HINARI discounts and waivers and eligibility is automatically checked during the submission process. We grant 100% waivers to articles whose corresponding authors are based in Group A countries and 50% waivers to those who are based in Group B countries as classified by Research4Life (see: https://www.research4life.org/access/eligibility/ ). This section has its own submission website at https://mc.manuscriptcentral.com/e-videos .
